# Efficacy of pudendal nerve block for alleviation of catheter-related bladder discomfort in male patients undergoing lower urinary tract surgeries: A randomized, controlled, double-blind trial

**DOI:** 10.1097/MD.0000000000008932

**Published:** 2017-12-08

**Authors:** Li Xiaoqiang, Zhang Xuerong, Liu Juan, Bechu Shelley Mathew, Yin Xiaorong, Wan Qin, Luo Lili, Zhu Yingying, Luo Jun

**Affiliations:** a West China Hospital, Sichuan University, Chengdu, China; b Department of Anesthesiology, Sun Yat-Sen Memorial Hospital, Sun Yat-Sen University; c Department of Pulmonary and Critical Care Medicine, West China Hospital, Sichuan University, Chengdu, China.

**Keywords:** catheter-related bladder discomfort, lower urinary tract surgery, pudendal nerve block, transurethral resection of bladder tumor, transurethral resection of prostate

## Abstract

**Background:**

Catheter-related bladder discomfort (CRBD) to an indwelling urinary catheter is defined as a painful urethral discomfort, resistant to conventional opioid therapy, decreasing the quality of postoperative recovery. According to anatomy, the branches of sacral somatic nerves form the afferent nerves of the urethra and bladder triangle, which deriving from the ventral rami of the second to fourth sacral spinal nerves, innervating the urethral muscles and sphincter of the perineum and pelvic floor; as well as providing sensation to the penis and clitoris in males and females, which including the urethra and bladder triangle. Based on this theoretical knowledge, we formed a hypothesis that CRBD could be prevented by pudendal nerve block.

**Objective:**

To evaluate if bilateral nerve stimulator-guided pudendal nerve block could relieve CRBD through urethra discomfort alleviation.

**Design and Setting:**

Single-center randomized parallel controlled, double blind trial conducted at West China Hospital, Sichuan University, China.

**Participants:**

One hundred and eighty 2 male adult patients under general anesthesia undergoing elective trans-urethral resection of prostate (TURP) or trans-urethral resection of bladder tumor (TURBT). Around 4 out of 182 were excluded, 178 patients were randomly allocated into pudendal and control groups, using computer-generated randomized numbers in a sealed envelope method. A total of 175 patients completed the study.

**Intervention:**

Pudendal group received general anesthesia along with nerve-stimulator-guided bilateral pudendal nerve block and control group received general anesthesia only.

**Main outcome measures:**

Incidence and severity of CRBD; and postoperative VAS score of pain.

**Results:**

CRBD incidences were significantly lower in pudendal group at 30 minutes (63% vs 82%, *P* = .004), 2 hours (64% vs 90%, *P < *.000), 8 hours (58% vs 79%, *P* = .003) and 12 hours (52% vs 69%, *P* = .028) also significantly lower incidence of moderate to severe CRBD in pudendal group at 30 minutes (29% vs 57%, *P < *.001), 2 hours (22% vs 55%, *P < *.000), 8 hours (8% vs 27%, *P* = .001) and 12 hours (6% vs 16%, *P* = .035) postoperatively. The postoperative pain score in pudendal group was lower at 30 minutes (*P* = .003), 2 hours (*P < *.001), 8 hours (*P < *.001), and 12 hours (*P < *.001), with lower heart rate and mean blood pressure. One patient complained about weakness in levator ani muscle.

**Conclusion:**

General anesthesia along with bilateral pudendal nerve block decreased the incidence and severity of CRBD for the first 12 hours postoperatively.

## 1 Introduction

### 1.1 Background and objectives

Catheter-related bladder discomfort (CRBD) secondary to an indwelling urinary catheter is defined as a burning sensation at urethra with an urge to void, urinary frequency and painful discomfort in the supra-pubic region.^[1–6]^ CRBD is a common complication often observed during the postoperative period in male patients undergoing transurethral resection of prostate (TURP) and transurethral resection of bladder tumor (TURBT); surgeries usually requiring a 20 or 22 Fr Foley's urethral catheter. These symptoms are resistant to conventional opioid therapy and this state is extremely distressing to the patient while in the postanesthesia care unit (PACU), decreasing the quality of postoperative recovery.^[1]^

The mechanism of CRBD is mediated by type 3 muscarinic receptor activation, which increases acetylcholine release and then causes the detrusor muscles of the bladder to contract involuntarily.^[7,8,31]^ Therefore, agents with anticholinergic properties including solifenacin,^[9]^ butylscopolamine^[3,10]^ oxybutynin^[2,11]^ and tolterodine,^[12]^ analgesics including tramadol4 and paracetamol,^[13]^ antiepileptics such as gabapentin^[14,15]^ and pregablin,^[16]^ anesthetics including ketamine and dexmedetomidine^[17,18]^ have been successfully studied for the prevention and treatment of CRBD. Nevertheless, these drugs when administered, generally can cause some side effects such as facial flushing, dry mouth, blurred vision and sedation.^[11,12,15,17]^

Pudendal nerve blocks (PNB) have been identified to be safe and effective in clinical applications^[31]^ for analgesia of labor and/or vaginal birth,^[19]^ vaginal repair,^[20]^ sphincterotomy,^[21]^ and the treatment of pudendal neuralgia.^[22]^ Previous studies have demonstrated that PNB is an effective anesthetic technique for reducing analgesic consumption and prolonging postoperative analgesia in young male patients undergoing circumcision and hypospadias.^[23,24]^

According to anatomy, the branches of sacral somatic nerves form the afferent nerves of the urethra and bladder triangle,^[7,31]^ which deriving from the ventral rami of the second to fourth sacral spinal nerves, innervating the urethral muscles and sphincter of the perineum and pelvic floor; as well as providing sensation to the penis and clitoris in males and females, which including the urethra and bladder triangle.^[25,26,31]^ Song et al^[27]^ performed autopsies on males and found that the dorsal nerve of the penis (DNP), the terminal branch of the pudendal nerve, innervates the membranous urethra in 53.3% of specimens.^[32]^ Thus based on this theoretical knowledge, we formed a hypothesis that CRBD could be prevented by PNB.

Thus the aim of this trial was to evaluate the effect of bilateral nerve-stimulator-guided pudendal nerve block in relieving CRBD by alleviating urethral discomfort, designed with the hypothesis that PNB has a CRBD-reductive effect, based on the anatomical and physiological proof for nerve innervation and with the support of clinical experience.

## 2 Methods

### 2.1 Ethics

This study was approved by the Clinical Trials and Biomedical Ethics Committee of West China Hospital of Sichuan University (Ethical code Ref: 2014 (188)) on January 30, 2015 and was registered in the Chinese Clinical Trial Registry (Registration number: Chi-CTR-IOR-15007559) on December 6, 2015. This trial was performed according to the principles of the Declaration of Helsinki (Edinburgh 2000 version). Written informed consent was obtained from each participant before enrolment.

### 2.2 Trial design

**Figure 1:**
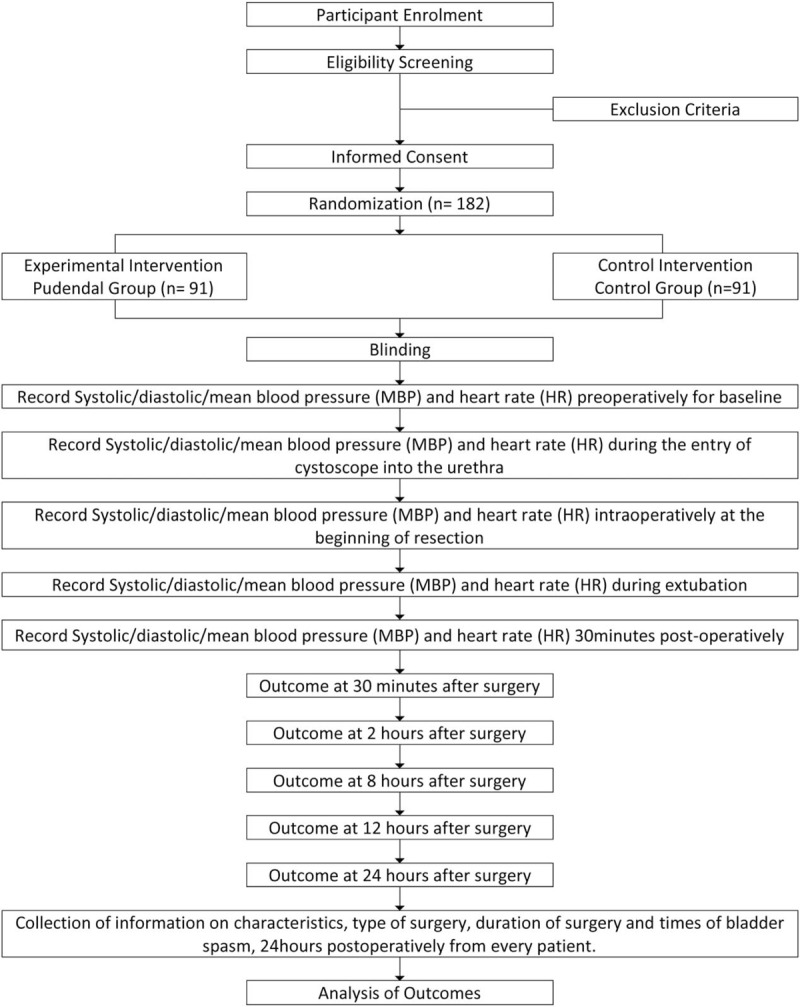
Trial scheme.

This trial is a randomized, parallel controlled, double-blinded, single-center study aimed at evaluating the effect of bilateral nerve-stimulator-guided pudendal nerve block in relieving CRBD by alleviating urethral discomfort. Participants were divided into 2 groups. (1) Pudendal group; receives bilateral nerve-stimulator-guided pudendal nerve block along with general anesthesia and (2) control group; receives only general anesthesia. The trial scheme is illustrated in Fig. 1.

### 2.3 Participants

**Figure 2:**
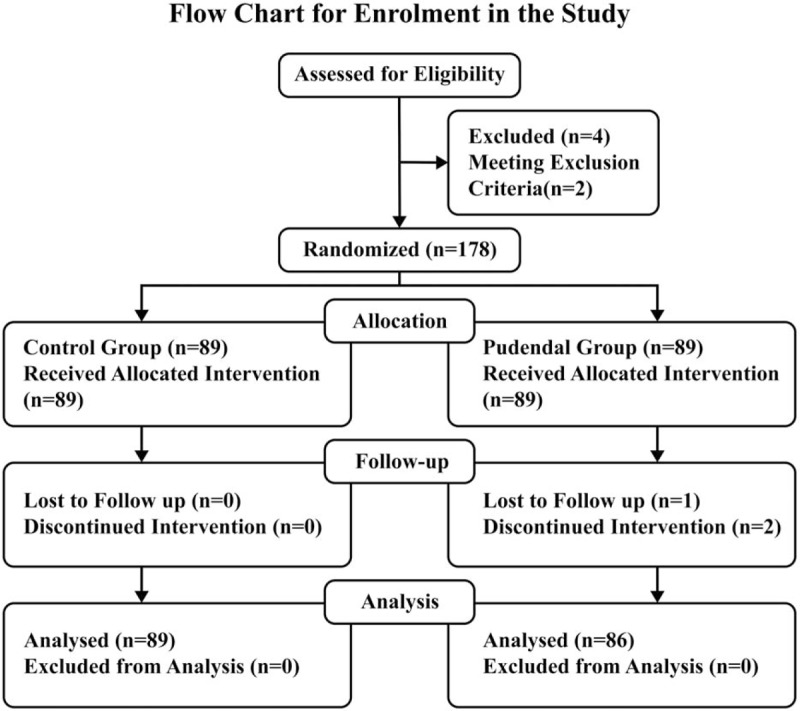
A flowchart for enrolment in the study.

Adult male patients undergoing elective trans-urethral resection of prostate (TURP) or trans-urethral resection of bladder tumor (TURBT) under general anesthesia, requiring intraoperative urinary catheterization were enrolled for the trial, from a single center (Fig. 2); West China Hospital of Sichuan University, Chengdu, China.

#### 2.3.1 Inclusion criteria

- Age ≥18 years
- American Society of Anesthesiologists (ASA) Physical Status I–III
- Without respiration or circulation disorders
- Without chronic pain.

#### 2.3.2 Exclusion criteria

- Body mass index (BMI) >40 kg/m^2^
- Allergy to local anesthetics
- Coagulopathy or bleeding disorder
- Pre-existing infection at the site of injection
- Mental disorders.

### 2.4 Interventions

Patients were monitored by noninvasive blood pressure, electrocardiography, and pulse oximetry after entering the operating room.

#### 2.4.1 Pudendal group

Anesthesia in the pudendal group was induced with midazolam 0.05 mg/kg, sufentanil 0.2 μg/kg, propofol 2 mg/kg and 4% sevoflurane in 100% oxygen for 2 to 4 minutes. After placement of a laryngeal mask, bilateral nerve-stimulator-guided pudendal nerve block was performed and the Sevoflurane concentration was decreased to 1.5% to 2% in oxygen with medical air (FiO_2_ 0.5), was maintained till the end of surgery.

Pudendal nerve block was done with the patient in lithotomy position at 2 separate puncture site located at 3 and 9 o’clock; approximately 3.5 to 4 cm from the center of the anus. Following aseptic preparation of the skin, a 100 mm nerve stimulator needle connected to a nerve stimulator was advanced to a depth of 2 to 3 cm perpendicularly to the skin with a stimulation current of 3.5 to 4.5 mA. The contraction of the anal sphincter was observed around here, indicating the stimulated inferior anal nerve. Then the needle was pushed progressively deeper until the pudendal nerve was stimulated, followed by up and down movement of penis and the contraction of the perineal muscles. As the best motor responses at a current of 3.0 to 3.2 mA is obtained, 0.5% ropivacaine solution was injected in 5 mL increments with negative blood aspiration before each increment accumulating to a total of 10 mL each in both sides. Two small pieces of surgical dressings were affixed at the 2 injection sites at the end of the block. Muscle relaxants were given only after a successful blockade.

#### 2.4.2 Control group

Induction and maintenance of anesthesia in the control group were analogous to the pudendal group, except for the administration of muscle relaxants.

For both the groups, patients were mechanically ventilated to keep the end-tidal carbon dioxide between 30 and 40 mm Hg. During the surgery, an increase in systolic blood pressure, heart rate, or both by more than 20% from baseline was defined as an insufficiency in anesthesia and analgesia, a bolus dose of 5 μg sufentanil was administered in case of increasing sevoflurane was not effective against this insufficiency. At the end of surgery, urinary catheterization was performed by the surgical team, using a 20 or 22 Fr Foley's catheter lubricated with tetracaine hydrochloride jelly. The catheter balloon was inflated with 30 ml distilled water and was fixed laterally to the thigh with tape, without any traction for free drainage into a urine bag. After the surgery, all patients received Neostigmine 1 mg and atropine 0.5 mg for antagonizing muscle relaxant. The patients were extubated and moved to the PACU after a satisfactory recovery from anesthesia.

### 2.5 Outcomes

#### 2.5.1 Primary outcomes

The primary outcome of this study was the intensity of bladder discomfort and postoperative pain and was assessed by a visual analog scale (VAS) ranging from 0 (no discomfort) to 10 (most severe discomfort). According to the given score, severity of CRBD is recorded as none (VAS 0), mild (VAS 1–3), moderate (VAS 4–7), and severe (VAS 8–10). Participants were well educated regarding the VAS once enrolled and asked to rate their pain level during the study, and also were educated about the CRBD and how to distinguish between postoperative pain and bladder discomfort and the numeric rating scale (NRS) during preoperative visits. Bladder discomfort and postoperative pain were evaluated at 30 minutes, 2, 8, 12, and 24 hours postoperatively by another anesthesiologist, unaware of patient group assignment.

#### 2.5.2 Secondary outcomes

Systolic/diastolic/mean blood pressure (MBP) and heart rate (HR) were recorded preoperatively for baseline, during the entry of cystoscope into the urethra, intraoperatively at the beginning of resection, during extubation and at 30 minutes postoperatively. These serve as the secondary outcomes.

Moreover, the following information: characteristics, type of surgery, duration of surgery, and times of bladder spasm were also collected 24 hours postoperatively from every patient.

### 2.6 Sample size

We conducted a pilot study to determine the sample size, and according to that study, 8 of 10 patients complained of CRBD postoperatively. Assuming that bilateral pudendal nerve block would reduce the incidence of CRBD from 80% to 60%, power analysis with α=0.05 and β=0.8 showed we would need to enroll 83 in each group for the results to be statistically significant. To make provision for a 10% dropout rate, we plan to include 91 patients per group.

### 2.7 Randomization

#### 2.7.1 Sequence generation

A set of random numbers for the allocation sequence were generated using a computerized SPSS software package (version 18; SPSS Inc., Chicago, IL).

#### 2.7.2 Allocation concealment mechanism

Sealed, opaque assignment envelopes were used for allocation concealment.

#### 2.7.3 Implementation

The included participants were randomly enrolled by sealed envelope and assigned to one of the 2 groups (pudendal group, or control group) in a 1:1 ratio. The patients were randomly divided into pudendal group or control group on the day of surgery by an anesthesiologist.

### 2.8 Blinding

An anesthesiologist blinded to the randomization opened the sealed envelope, performed PNB, and did not involve in the perioperative and postoperative management of the patients. Data were collected during the intraoperative period and postoperative follow-up by another anesthesiologist, blinded to the patient group assignment. Moreover; patients, surgeons, and assisting personnel were also made unaware of the patient group assignment. Two small pieces of surgical dressings were placed at the symmetrical sites for all the randomized patients, making sure every patient looks consistent.

Unblinding was permissible only if required under emergency conditions that compromise the health and safety of subjects; in which case the surgeons and the attending anesthesiologists would be informed about the subjects, thereby terminating the study for that patient.

### 2.9 Statistical methods

Statistical analysis was performed with the SPSS (version 18; SPSS, Inc., Chicago, IL) and SAS (version 9.3; SAS Inst., Inc., Cary, NC). The incidence and severity of CRBD was compared using Chi-square test or Fisher's exact test (if cell size ≤ 5). Postoperative pain on a VAS scale was analyzed by the Mann–Whitney test. The Bonferroni correction was used to counteract the problem of multiple comparisons in the above 2 methods, and each individual hypothesis would test at α=0.05/5 = 0.01 because of the 5 measured times. Mean blood pressure and heart rate between the 2 groups were analyzed by repeated measures ANOVA followed by post-hoc analysis.

## 3 Results

### 3.1 Participant flow

In the study, 182 patients were evaluated for inclusion (Fig. 1.). Four patients were excluded from the study [exclusion criteria (n = 2), refusal (n = 2)] thus178 patients were included and randomized. Three patients from pudendal group were considered as dropped from the study as 1 lost to follow-up and 2 patients could not differentiate sensing pain and temperature between perineum and inner thigh regions. Therefore, 175 patients completed the study for further statistical analysis (89 in the control group and 86 in pudendal group). Table 1 shows data on patients, surgeries and anesthesia.

**Table 1:**
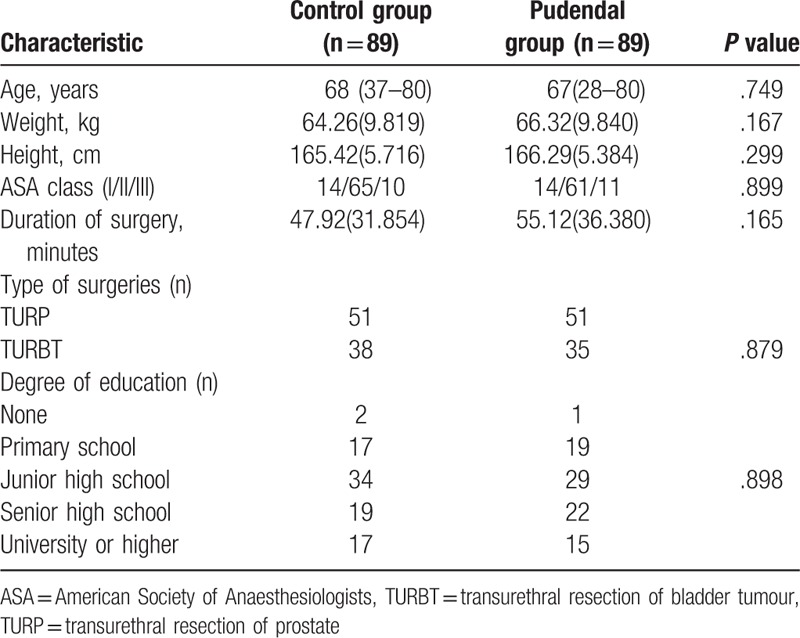
Characteristics of participants in this study.

### 3.2 Recruitment

This trial was conducted from October 2015 to April 2017. The trial was ended upon fulfilling the required sample size.

### 3.3 Baseline data

#### 3.3.1 Numbers analyzed

A total of 86 cases were analyzed for pudendal group and 89 cases were analyzed for control group and by original assigned groups.

### 3.4 Outcomes and estimation

**Figure 3:**
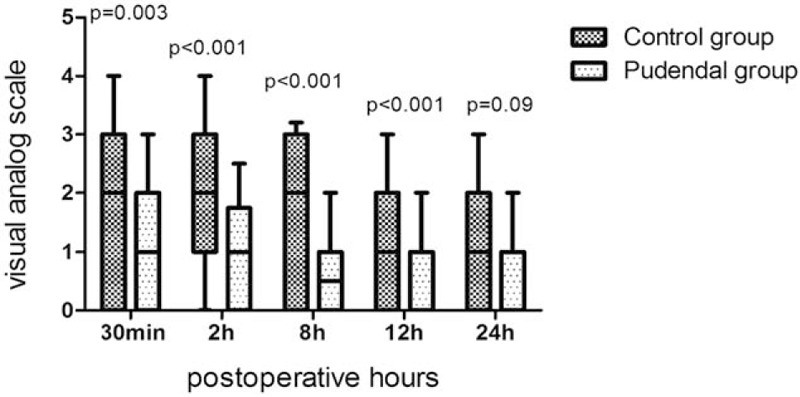
Visual analog scale pain scores during the postoperative 24 hours. The box-plot shows the median with interquartile range, 10th and 90th percentiles (whiskers).

The incidence of postoperative CRBD at 30 minutes (63% vs. 82%, *P* = 0.004), 2 hours (64% vs. 90%, *P < *.000) and 8 hours (58% vs. 79%, *P* = .003) was lower in the pudendal group than that in the control group (Table 2.). The incidence of moderate to severe CRBD was also significantly lower in the pudendal group than that in the pudendal group at 30 minutes (29% vs. 57%, *P < *.001), 2 hours (22% vs. 55%, *P < *.000), and 8 hours (8% vs. 27%, *P* = .001) postoperatively. After that time, the incidence and severity of CRBD were comparable between the 2 groups at 12 and 24 hours postoperatively (*P* > .01). The postoperative pain score was reduced in the pudendal group when compared with the control group at all-time points (*P < *.01) except 24 hours postoperatively (Fig. 3).

**Table 2:**
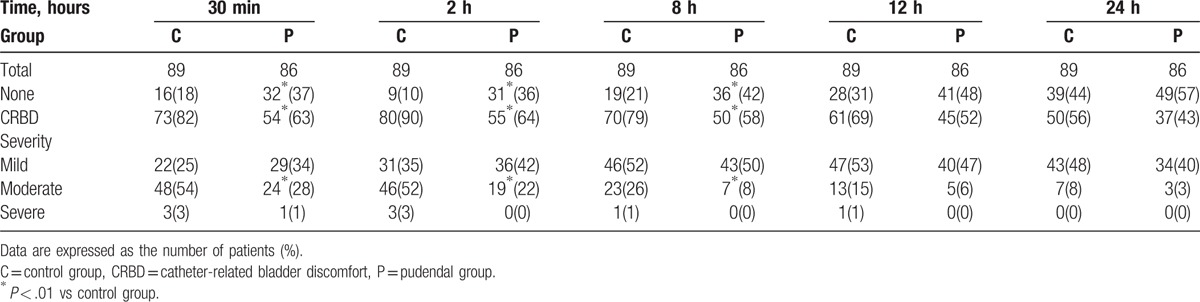
Incidence and severity of catheter-related bladder discomfort (CRBD).

**Figure 4:**
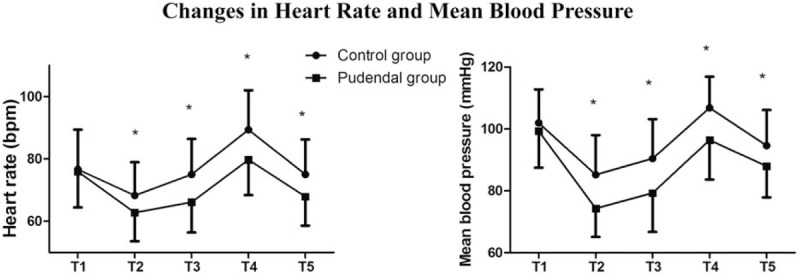
Changes in HR (*P* = .000) and MBP (*P* = .000) in the 5 times are significant. Compared with T1 (baseline), the following 4 times as T2 (during the entry of cystoscope into the urethra), T3 (intraoperatively at the beginning of resection), T4 (during extubation), T5 (30 minutes postoperatively) are significantly different in both HR and MBP. ^∗^*P* < .05. HR = heart rate, MBP = mean blood pressure.

In the pudendal group, HR and MBP were lower; during the entry of cystoscope into the urethra (T2); at the beginning of resection (T3); during extubation (T4); and 30 minutes postoperatively (T5) (*P < *.05) (Fig. 4).

### 3.5 Harms

One patient complained about weakness in levator ani muscle.

## 4 Discussion

### 4.1 Limitations

The shortcomings of this study are: firstly, the surgeries are involved in the operation of the prostate and bladder, and are operated through the urethra, the 2 types of surgery itself may cause postoperative bladder and urethral discomfort. Then the scores of those patients who cannot distinguish between the sources of discomfort may be biased to some extent; Furthermore, throughout the trial, we did not get same group of surgeons to do all the surgeries, different surgeons have different habits and different surgical procedures, this can also cause a certain degree of interference.

Potential complications of pudendal nerve block include local anesthetics poisoning, local anesthetic allergy, local anesthetic extends to the sciatic nerve region leading to sciatic nerve block, sciatic fossa hematoma, infection or deep abscess, and pudendal nerve injury. Thus the implementation of pudendal nerve block have certain requirements, such as the need to be under the guidance of a nerve stimulator or ultrasound for completion, the need to spend some time before the operation put the patient into lithotomy position. In order to avoid discomfort and embarrassment to the patient, the pudendal nerve block under the guidance of nerve stimulation is usually carried out after general anesthesia, and could not complete in the awake state.

Yet in our study there was 1 patient complaining about lack of strength in his levator ani muscle. However, it is possible to reduce the incidence of motor nerve blockade if the concentration of the anesthetic is reduced.

### 4.2 Generalizability

Aissaoui et al^[29]^ considers that pudendal nerve block is a simple and useful technology, and with the full implementation of 10 cases one can fully grasp the technology. In the present study, all the block operations were done by the same anesthesiologist using the nerve stimulation machine to ensure the homogeneity of the block effect. The use of 0.5% ropivacaine in the study of nerve block in the postoperative follow-up found no nerve block complications, such as bleeding, hematoma, puncture pain, infection and so on.

In our center, the medical staff used compound lidocaine cream or tetracaine hydrochloride jelly in the catheter and urethral mouth before clinical catheterization, to reduce urethral injury and the stimulation by catheter on the urethra and bladder. But tetracaine and lidocaine are short-acting mucosal surface aesthetic and their maintenance time is short. The use of long-acting local anesthetics (ropivacaine) of pudendal nerve block can last for more than 10 hours.

Using ultrasound guiding would be more convenient and easy. So this technique of pudendal nerve block can be improved in many ways in clinical practice and can be performed faster.

### 4.3 Interpretation

In patients undergoing TURBT and TURP, 20 or 22 Fr Foley's catheters should be placed after surgery, and the incidence of CRBD is greater and more severe if the catheter is comparatively thicker.^[1]^ In order to protect the patient's privacy, surgical patients often require a urinary catheter after anesthesia, as a result after waking up, the ureter stimulation can suddenly induce dysphoria, tossing and turning, and even make the incision bleed or rupture. Thereby prolonging the patient's hospital stay and increasing medical expenses.

For penile surgery, nerve block can be done through the dorsal penile nerve block on the corpus cavernosum and urethra. The urethral stricture caused by traumatic injury often causes a urine bulb during pelvic fracture, there is no good method for analgesia after surgery, and the pudendal nerve block should serve as an effective method of postoperative analgesia.

Urethral and bladder mucosa is very rich in nerve endings, the mucous membrane can feel various stimuli and send through the afferent nerve to reach the nerve center, causing a variety of bladder and urinary tract irritation.^[8,30]^ Causes of CRBD may be due to the stimulation of urethra by the urinary catheter as well as by the stimulation of the bladder trigone area by the water sac of the tube. In theory, CRBD can be reduced by reducing the irritation of the urethra and bladder mucosa by blocking the nerve endings of the mucosa. The thicker the urinary catheter, the greater the stimulation of the urethra, which is why we included these 2 types of surgical patients in this study. Physiologically speaking, the urethra is longer in male patients than that in females, which is about 16 to 20 cm. Thus the male patients usually have a higher incidence of CRBD than the females, and it is proved that the stimulation by the urinary catheter is an important cause of CRBD 1. As per the hypothesis before this study, we proved that pudendal nerve block can reduce the incidence of postoperative CRBD and CRBD severity. The reason may be the pudendal nerve block obstructs the stimulation from the urinary tract of male patients caused by the catheter.

Thus through this study, we were able to find that the application of bilateral nerve-stimulator-guided pudendal nerve block is helpful in the alleviation and reduction of incidences of CRBD in patients undergoing lower urinary tract surgeries. It is also a method with lesser side effects when compared to the usage of anticholinergic and other drugs for postoperative analgesia.

### 4.4 Uncited reference

^[28]^.
